# Supplementary material for: Deep Transcranial Magnetic Stimulation Affects Gut Microbiota Composition in Obesity: Results of Randomized Clinical Trial
Source: Int J Mol Sci. 2021 Apr 29;22(9):4692. doi: 10.3390/ijms22094692 (PMC8125086; doi:10.3390/ijms22094692)
Supplement: Supplementary file 1 [file ijms-22-04692-s001.zip › ijms-1158896-supplementary.pdf]

SUPPLEMENTARY MATERIAL

Deep Transcranial Magnetic Stimulation affects gut microbiota composition in obesity: results of randomized clinical trial.

Anna Ferrulli[1,2], Lorenzo Drago[2], Sara Gandini[3], Stefano Massarini[1], Federica Bellerba[3], Pamela Senesi[1,2], Ileana Terruzzi[1,2] and Livio Luzi[1,2,*]

[1] Department of Endocrinology, Nutrition and Metabolic Diseases, IRCCS MultiMedica, Sesto San Giovanni (MI), Italy;
[2] Department of Biomedical Sciences for Health, University of Milan, Milan, Italy;
[3] Department of Experimental Oncology, European Institute of Oncology IRCCS, Milan, Italy.

* Correspondence: Livio Luzi, MD; Head, Department of Endocrinology, Nutrition and Metabolic Diseases IRCCS MultiMedica; Via Milanese, N. 300, 20099 Sesto San Giovanni (MI), Italy
E-mail: livio.luzi@unimi.it; livio.luzi@multimedica.it
ORCID: 0000-0003-3183-0552
Tel: +39 02.85994286
Mob: +39 340 8435693

Stimulation procedure

Before each dTMS session, the Resting Motor Threshold (RMT) was determined over the left primary motor cortex. The optimal spot on the scalp was localized to stimulate the right abductor pollicis brevis muscle, and the RMT was defined by delivering single stimulations, applying one pulse every 5 seconds to the motor cortex, and gradually decreasing intensity. The RMT was defined as the stimulation with the lowest required intensity to cause the right thumb to move. Once the RMT was defined, the stimulation could begin.

High-frequency sessions consisted of 80 trains of 18 Hz, each lasting 2 seconds, with an intertrain interval of 20 seconds. The HF treatment duration was 29.3 minutes with 2880 pulses in total. Low-frequency sessions consisted of 4 trains of 1 Hz, each lasting 10 minutes, with an intertrain interval of 1 minute. The LF treatment duration was 43 minutes with 2400 pulses in total. The Sham treatment was performed by a sham coil located in the same case of the real coil, producing similar acoustic artefacts and scalp sensations, inducing only negligible electric fields in the brain. In all groups receiving the real treatment, the stimulation was performed with an intensity of 120% of the RMT.

Table S1. All the variables analyzed at baseline and after 5 weeks of treatment, in the 3 treatment groups, including anthropometric measurements, bio-markers and gut microbiota phyla and genera.

	**HF**		**LF**		**SHAM**	
	T0	T2	T0	T2	T0	T2
Body weight (Kg)	106.8±6.0	103.1±6.3	111.1±4.6	108.9±4.6	95.4±3.7	95.3±4.6
BMI (Kg/m^2)	37.3±2.0	31.8±4.3	40.7±2.1	39.9±2.2	35.5±1.1	35.7±1.0
Glucose (mg/dL)	95.0±5.9	91.7±5.0	103.2±14.9	97.7±13.3	95.9±6.5	90.9±3.4
Cholesterol (mg/dL)	208.9±11.3	203.1±12.3	179.0±16.4	170.7±17.6	189.0±7.2	178.6±8.1
Triglycerides (mg/dL)	146.1±4.7	145.6±16.9	143.7±35.1	147.3±40.4	103.1±17.1	99.1±10.0
Insulin (μU/mL)	27.1±8.1	19.7±4.1	21.6±2.6	19.3±3.6	20.1±6.7	14.2±2.9
HOMA-IR	7.2±2.5	4.8±1.2	5.6±1.1	5.0±1.6	4.8±1.7	3.2±0.7
Glucagon (pg/Ml)	40.7±3.7	35.5±4.1	34.4±2.2	39.5±3.4	43.2±4.9	43.3±4.0
Fructosamine (μmol/L)	243.9±10.0	234.3±11.0	221.7±8.8	222.0±11.8	234.1±14.1	236.9±3.6
Glycated hemoglobin (mmol/mol)	36.9±1.5	35.0±1.2	41.0±7.1	41.8±8.1	34.8±2.8	32.0±2.2
Ghrelin (ng/mL)	31.1±3.5	7.3±2.0	13.0±3.6	9.5±2.2	7.9±2.9	8.2±2.0
Leptin (ng/mL)	64.7±13.1	48.9±22.3	83.4±22.5	57.6±13.1	108.3±49.9	56.7±16.5
Epinephrine (pg/mL)	1356.0±261.8	1622.4±471.3	996.7±151.1	824.5±50.6	1487.7±446.2	1613.8±410.9
Norepinephrine (ng/mL)	4.69±0.74	1.81±0.68	4.7±0.9	3.1±0.7	4.2±0.9	3.4±1.5
β-endorphin (ng/mL)	0.71±0.12	0.65±0.10	0.74±0.21	0.60±0.20	0.55±0.09	0.50±0.10
TSH (μUI/mL)	2.4±0.3	2.6±0.4	2.38±0.7	1.82±0.5	3.2±0.7	2.7±0.6
Cortisol (μg/dL)	0.43±0.06	0.39±0.06	0.40±0.06	0.30±0.10	0.35±0.05	0.30±0.00
FM (%)	46.6±2.4	46.2±2.1	46.6±2.5	46.9±2.5	45.7±2.3	45.2±2.2
RQ	0.89±0.02	0.85±0.02	0.89±0.02	0.84±0.01	0.85±0.03	0.90±0.00
REE (%)	101.0±3.0	86.6±3.7	94.0±6.2	88.4±2.1	93.1±5.0	89.0±3.0
Firmicutes (%)	25.1±3.9	25.3±3.2	18.6±5.4	16.7±4.3	18.9±1.9	18.6±1.9
Bacteroidetes (%)	25.9±2.8	20.0±3.5	38.8±9.0	30.4±6.2	26.6±1.4	25.8±1.4
Firmicutes:Bacteroidetes	1.83±1.07	2.50±1.05	0.81±0.32	0.71±0.20	0.72±0.08	0.71±0.11
READS ABUNDANCE						
Alistipes	1490.7±656.7	3777.8±1137.8	752.9±505.5	339.7±196.8	1779.8±640.0	1510.4±444.3
Bacteroides	12570.1±2826.3	16716.7±2550.1	23000.3±13177.8	11477.2±4387.2	23452.9±5971.6	27274.4±5753.0
Bilophila	4543.0±1613.1	6810.2±3138.2	3178.2±1479.4	2587.5±1357.7	896.8±477.9	2856.1±800.2
Clostridium	1720.0±806.8	2547.2±822.1	89.7±52.5	540.0±225.0	540.4±241.6	165.9±69.1
Coprococcus	39.5±16.5	122.4±64.7	90.7±48.6	50.4±27.8	88.4±45.4	248.8±168.2
Eubacterium	1883.3±529.2	1189.7±287.7	1072.9±713.1	723.8±523.7	1827.3±774.0	806.5±459.5
Faecalibacterium	3105.4±1425.5	7897.3±2314.3	2869.4±1285.3	5927.1±2202.3	4471.9±1449.9	5704.9±948.8
Gemmiger	241.7±116.9	422.0±166.0	155.9±18.0	159.1±54.3	80.5±41.6	1074.1±494.4
Lactobacillus	260.1±115.8	59.7±33.1	777.1±445.6	574.8±348.7	155.7±112.0	511.1±324.7
Odoribacter	38.6±20.0	261.7±114.9	95.2±37.8	68.0±50.0	201.7±65.1	368.4±106.2
Parabacteroides	365.1±198.1	697.4±189.6	1240.9±938.0	730.8±444.0	995.7±383.0	869.7±278.5
Parasutterella	226.4±90.0	237.4±100.2	1320.3±792.6	2143.8±1834.4	469.4±268.8	1326.9±792.2

Phascolarctobacterium	4436.9±1969.1	5373.7±2264.8	4928.8±4628.2	4962.5±3862.0	2263.7±1062.8	8441.6±3259.3
Roseduria	2966.1±905.08	1757.3±1162.1	509.9±145.7	690.7±333.7	887.7±423.0	553.7±208.9
Ruminococcus	3891.0±1356.1	3394.9±1645.8	245.6±113.0	339.9±211.2	3034.3±869.2	1302.1±562.4
Streptococcus	430.0±270.9	385.9±253.3	49.0±24.3	1044.8±749.5	589.7±195.3	474.3±321.3
Sutterella	5235.6±2340.0	6319.4±2763.0	3362.9±233.9	3482.9±2893.9	3215.5±1663.1	6099.7±2303.6

Table 1. Data are shown as mean ± standard error of the mean (SEM) at baseline (T0) and at the last (15th) dTMS session (T2).

Abbreviations: HF=High Frequency; LF=Low Frequency; BMI=Body Mass Index; FM=Fat Mass; REE= Resting Energy Expenditure; RQ=Respiratory Quotient; HOMA-IR= HOmeostatic Model Assessment-Insulin Resistance; TSH=Thyroid Stimulating Hormone.

Table S2: Intercept and beta coefficient of linear regression models of norepinephrine changes and genera variations in the HF group, after 5 weeks of dTMS treatment.

Eubacterium	*Parasutterella*	*Bacteroides*
y = 0.3389x - 0.6642	y = 5.4998x + 4.3571	y = -2.1564x - 0.3501
